# Laboratory and Field Evaluations of the GeoAir2 Air Quality Monitor for Use in Indoor Environments

**DOI:** 10.4209/aaqr.220119

**Published:** 2022-06-01

**Authors:** Dillon Streuber, Yoo Min Park, Sinan Sousan

**Affiliations:** 1 Environmental Health Sciences Program, Department of Health Education and Promotion, College of Health and Human Performance, East Carolina University, Greenville, NC 27858, USA; 2 Department of Geography, Planning, and Environment, East Carolina University, Greenville, NC 27858, USA; 3 Department of Public Health, Brody School of Medicine, East Carolina University, Greenville, NC 27834, USA; 4 North Carolina Agromedicine Institute, Greenville, NC 27834, USA

**Keywords:** Particulate matter, PM_2.5_, Indoor air quality, SPS30 sensor, PM humidity effects

## Abstract

Low-cost aerosol sensors open routes to exposure assessment and air monitoring in various indoor and outdoor environments. This study evaluated the accuracy of GeoAir2––a recently developed low-cost particulate matter (PM) monitor––using two types of aerosols (salt and dust), and the effect of changes in relative humidity on its measurements in laboratory settings. For the accuracy experiments, 32 units of GeoAir2 were used, and for the humidity experiments, 3 units of GeoAir2 were used, alongside the OPC-N3 low-cost sensor and MiniWRAS reference instrument. The normal distribution of slopes between the salt and dust aerosols was compared for the accuracy experiments. In addition, the performance of GeoAir2 in indoor environments was evaluated compared to the pDR-1500 reference instrument by collocating GeoAir2 and pDR-1500 at three different homes for five days. For salt and dust aerosols smaller than 2.5 μm (PM_2.5_), both GeoAir2 (r = 0.96–0.99) and OPC-N3 (r = 0.98–0.99) were highly correlated with the MiniWRAS reference instrument. However, GeoAir2 was less influenced by changes in humidity than OPC-N3. While GeoAir2 reported an increase in mass concentrations ranging from 100% to 137% for low and high concentrations, an increase between 181% and 425% was observed for OPC-N3. The normal distribution of the slopes for the salt aerosols was narrower than dust aerosol, which shows closer slope similarities for salt aerosols. This study also found that GeoAir2 was highly correlated with the pDR-1500 reference instrument in indoor environments (r = 0.80–0.99). These results demonstrate potential for GeoAir2 for indoor air monitoring and exposure assessments.

## 1 INTRODUCTION

The United States Environmental Protection Agency (U.S. EPA) identifies particulate matter (PM) with a diameter of 2.5 μm or smaller in size (PM_2.5_) as one of the criteria air pollutants. PM_2.5_ is of interest for its health effects and its ability to form from primary and secondary emissions from reactions between other pollutants present in the air (U.S. EPA, 1990). Lifelong exposure to PM can bring a host of negative health effects from silicosis related to silica exposure and lung cancer linked to tobacco smoke (Collins *et al*., 2005; Slezakova *et al*., 2009). In addition, elevated PM_2.5_ concentrations are linked to mortality from heart disease, stroke, lung cancer, chronic obstructive pulmonary disease and acute respiratory infections (Brook *et al*., 2010; Hansel *et al*., 2016; Li *et al*., 2017; Huang *et al*., 2019; Kim *et al*., 2020). PM_2.5_ is generated from outdoor sources, such as mobile emissions, industrial activities, coal and biomass burning, wildfires, dust and sea spray aerosols (Calvo *et al*., 2013), and indoor sources, such as cooking practices, smoking, incense, candles, fireplace burning, and outdoor infiltration (Owen *et al*., 1992; Marć *et al*., 2018). It has been suggested that indoor air quality may be of greater health risk than outdoor exposure (Cincinelli and Martellini, 2017), and indoor exposure to PM can exceed 500 μg m^−3^, causing serious health problems (Diapouli *et al*., 2008). Despite its adverse health impacts, environmental, public health policies and research efforts have historically focused on outdoor air quality due to its environmental effect, and relatively limited attention has been paid to indoor air quality (WHO, 2010). However, in the U.S and other industrialized countries, most people spend more than 80% of their time indoors (Franklin, 2007), so developing methods/technologies to assess indoor exposure accurately and react accordingly is critical.

Over the past few years, significant improvements in aerosol sensor technologies have allowed researchers to capture exposure in indoor environments for better accuracy. The air sensor technologies come in various forms, including real-time optical particle counters (OPCs), photometers, and spectrometers (Sousan *et al*., 2016b). OPCs use light scattering technology to detect particles of different sizes and calculate mass concentration compared to photometers that determine the mass concentration based on the reflected light from a bulk of particles. However, their high prices ($3,000–$100,000) have relegated these devices to research and industrial application. These limitations create a large monetary barrier for communities and individuals interested in providing representation for themselves and those around them. As a result, many individuals go unrepresented as to their exposure (Jiao *et al*., 2015). There has been a trend in developing low-cost aerosol sensors to open the description of exposures to individuals to represent their indoor air quality (Popoola *et al*., 2018).

Low-cost OPCs can provide accessibility to air quality representation to individuals that cannot afford the higher-cost sensors. An added benefit is the tendency to be lightweight and smaller in size, providing the opportunity to deploy these sensors indoors for personal use compared to their higher-cost counterparts (Sousan *et al*., 2021). These low-cost sensors include the OPC-N3 (Alphasense, Essex, Great Notley, United Kingdom) and the SPS30 (Sensiron AG, Stäfa, Switzerland). Tryner *et al*. (2020) tested the SPS30 and showed consistent readings for dust PM_2.5_ concentrations over the long periods and higher precision than other low-cost OPCs. Sousan *et al*. (2021) reported a high correlation (r = 0.99) for the SPS30 and OPC-N3 compared to the reference instrument in environmental and occupational settings with a moderate bias for salt aerosol PM_2.5_ measurements after testing three pairs of SPS30 and OPC-N3. Other low-cost OPCs include the Plantower PMS5003, Dylos, Honeywell HPMA115S0 and PM Nova which have been evaluated in laboratory and field settings (Sousan *et al*., 2016b; Levy Zamora *et al*., 2019; Hong *et al*., 2021; Dubey *et al*., 2022).

Although low-cost OPCs are promising, environmental conditions can affect their classification of particles (Crilley *et al*., 2018, 2020). For example, in high relative humidity conditions, hygroscopic growth can sheathe a particle in a layer of water (Svenningsson *et al*., 1992). However, the true size does not matter in inhalation. This overestimated size is the actual size that people inhale in humid conditions. Therefore, OPCs can misclassify a particle as larger than its true size, causing it to overestimate the mass of detected aerosol (Crilley *et al*., 2018). Although previous studies have performed experiments on various low-cost sensors alongside the SPS30 to determine the effect of changing relative humidity on reported PM_2.5_ concentration against reference instruments such as a TEOM (Wang *et al*., 2021), they have not derived correction equations for the SPS30 from this change in relative humidity with mass concentration. To support assessments of disease burden from indoor aerosols, reliable and accurate indoor air quality monitoring should be preceded.

The objectives of this study were 1) to determine the accuracy of 32 SPS30’s built in the GeoAir2––a low-cost, GPS-enabled, portable air-monitoring platform (Park *et al*., 2021)––using salt and dust aerosols in laboratory settings; 2) to identify the effect of relative humidity on GeoAir2’s PM_2.5_ mass concentrations using salt aerosol in laboratory settings; and 3) to evaluate the GeoAir2’s real-time indoor PM_2.5_ readings in three individuals’ homes compared to those of a filter-corrected real-time reference instrument to determine the accuracy in indoor environments. This study provides the required assessment for the GeoAir2 and necessary data that position the instrument as a viable air quality monitor for indoor settings and possible outdoor use.

## 2 MATERIALS AND METHODS

The specifications and differences for the low-cost and reference instruments are shown in Table 1.

### 2.1 Low-Cost Sensors

#### 2.1.1 GeoAir2

The GeoAir2 (East Carolina University, Greenville, NC, USA) is a recently developed portable air-monitoring platform that provides geo-referenced real-time PM_2.5_ concentrations by combining air sensors and a GPS module. The monitor costs $250–$350 depending on the units ordered (Park *et al*., 2021). It uses the SPS30 for PM_2.5_ monitoring, includes volatile organic compound (VOC) and hydrogen H_2_-based carbon dioxide (CO_2_) sensors, and provides temperature, humidity, time, and GPS logging. The SPS30 has five bins from 0.3 μm to 10 μm, converted to number counts and mass concentrations using proprietary equations (Sensirion, 2020). Compared to other commercially available devices existing in the market, the benefit of using GeoAir2 for this study is that it can be used in any indoor place with limited or no Wi-Fi access because it does not require Wi-Fi access or rely on smartphone applications to transfer data. In addition, encrypted data files are stored on a microSD card inside the platform.

#### 2.1.2 OPC-N3

The OPC-N3 costs $500 and features 24 bins from 0.35 μm to 40 μm which are converted into number counts and mass concentrations with algorithms developed by the company to measure mass concentrations of particles from 0 to 2000 μg m^−3^ (Alphasense, 2019). The device also features built-in temperature and relative humidity sensors and an internal fan. However, it does not feature an internal battery, so power must be supplied. The OPC-N3 specifications are included in Table 1, and its small form-factor allows for easy deployment. However, the device requires an external power source and a dedicated computer to retrieve time-stamped data, where the OPC-N3 does not have an internal clock (Alphasense, 2019).

### 2.2 Reference Instruments

#### 2.2.1 pDR-1500

The personal DataRAM (pDR-1500, Thermo Fisher Scientific, Waltham, Massachusetts, USA) is a photometer that uses a cyclone to measure particulate mass concentration at a specific particle size (Thermo, 2016). The device is a reference instrument, providing a filter to correct real-time data. The pDR is equipped with a 37 mm fiberglass filter (Whatman, CATNon.1827–037, Maidstone, United Kingdom) for particle collection that can then be removed and weighed for gravimetric analysis.

#### 2.2.2 MiniWRAS

The GRIMM Mini Wide Range Aerosol Spectrometer (MiniWRAS, GRIMM Aerosol Technik Ainring GmbH & Co. KG, Ainring, Germany) uses a corona charge to measure particles smaller than 0.25 μm by supplying unipolar ions to charge the aerosol and measure the charge using a Faraday Cup Electrometer. Particles between 0.25 and 35 μm are measured using an optical sensor (GRIMM, 2021). The device reports number concentrations in 41 bins, including PM_2.5_ mass concentrations.

### 2.3 Experimental Setup to Evaluate the Accuracy, Bias and Precision

#### 2.3.1 Chamber description

**Fig. 1.**
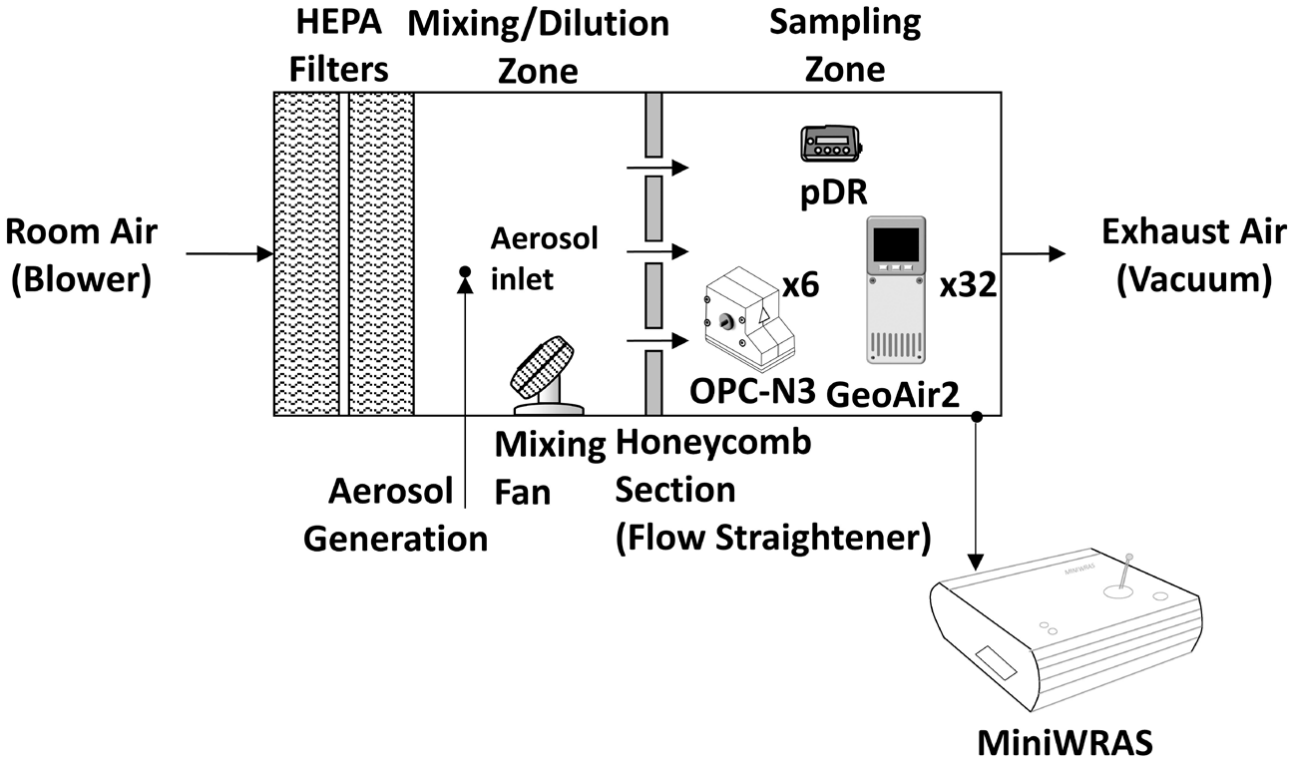
The experimental chamber used to test the sensors for salt and dust particulate matter detection.

The experiments were performed inside a controlled, airtight, plexiglass exposure chamber with dimensions 1.82 m × 0.66 m × 0.66 m (L × W × H) as shown in Fig. 1. This chamber was split into a mixing/dilution zone and a sampling zone. Both zones were split by a honeycomb straightening section (AS100, Rusken, Grandview, MO, USA). The mixing/dilution zone measured 0.61 m × 0.61 m × 0.66 m (L × W × H) with an inlet on both the bottom and side to introduce generated aerosol. Mixing was accomplished with two small fans in this zone. Particle-free air was supplied for the mixing process by two High-Efficiency Particulate Air (HEPA) filters (99.99% efficiency rating each). The sampling zone measured 0.61 m × 0.61 m × 0.66 m (L × W × H), leading to a vacuum outlet that exhausted air through two HEPA filters. A valve on the exhaust outlet allowed adjustment of the flow rate through the sampling zone. During the experiment, 32 GeoAir2, 6 OPC-N3’s, and the pDR-1500 (equipped with a 2.5 μm cyclone − 50% cut-point) were located directly within the sampling zone of the chamber. The MiniWRAS was placed outside the chamber with a sampling probe inside the sampling zone.

#### 2.3.2 Aerosol generation

Salt and dust aerosols were generated using different generation methods. Salt was chosen for its wide use and relative safety to help evaluate the GeoAir2 units (Sousan *et al*., 2016b). Salt aerosol was generated with the Aerogen nebulizer (Aerogen, Galway, Connacht, Ireland) using a 2% (by wt.) solution of NaCl. A mass flow controller (Cole-Parmer 32907–73, Antylia Scientific, Vernon Hills, Illinois, USA) supplied particle-free air from a five-stage desiccant into a silica column. Then, the salt aerosol was passed through a silica column to remove moisture, and dry salt particles entered the mixing zone of the chamber, where particle-free air was introduced to achieve the desired steady-state concentrations.

The second aerosol chosen was Arizona Road Dust (ARD; PTI ID: 13328B, Powder Technology Inc, MN, USA) due to its wide use and similarity to coarse mineral dust found in indoor settings. The aerosol was generated utilizing the Vilnius Aerosol Generator (VAG, CH Technologies, Westwood, New Jersey, USA). The VAG dispenses dry powder to produce aerosol concentrations from 1 to 2500 mg m^−3^. ARD was loaded into the device 1.33 g at a time, and once assembled, particle-free air was supplied and controlled by the same mass flow controller mentioned above. This mixture was supplied directly into the mixing zone of the chamber with particle-free air to achieve the desired steady-state concentrations.

The GeoAir2, OPC-N3, and pDR-1500 were set to record real-time measurements with one-second frequency, while the MiniWRAS recorded every one minute. The pDR-1500 also provided 37 mm filters pre- and post-weighed before and after the experiments using a Mettler Toledo microbalance (Model: XPR26DR, Columbus, Ohio, USA) and anti-static kit with a large U-electrode (Model: 63052302, Mettler Toledo, Columbus, Ohio, USA). The pDR-1500 operated at a flow rate of 1.52 LPM across all experiments.

All sensors were operated in particle-free air to achieve a concentration of 0 μg m^−3^ for five minutes before steady-state concentrations were achieved at 10, 20, 30, 40, 50, 100, 200, 300, 400, and 500 μg m^−3^. The laboratory experiments were performed to simulate indoor conditions at low (up to 50 μg m^−3^) and high (up to 500 μg m^−3^) concentrations. Calibrating sensors at low and high concentrations have shown calibration differences, which justifies performing these separately (Sousan *et al*., 2021). Since the World Health Organization guidelines for air quality recommend a 25 μg m^−3^ 24-hour mean for PM_2.5_, twice the value of 50 μg m^−3^ was chosen as the upper limit for low concentration (WHO, 2010). The high concentration was based on the literature previously mentioned in the Introduction (Diapouli *et al*., 2008). The pDR-1500 was used to monitor the steady-state concentrations achieved with salt and ARD aerosol. Aerosol size distribution of the SPS30 inside the GeoAir2, OPC-N3, and MiniWRAS were measured for salt and ARD aerosols in a previous study conducted by the author’s (Sousan *et al*., 2021), therefore, these measurements were not included in this work.

### 2.4 Experimental Setup to Determine the Effect of Relative Humidity

#### 2.4.1 Chamber description

**Fig. 2.**
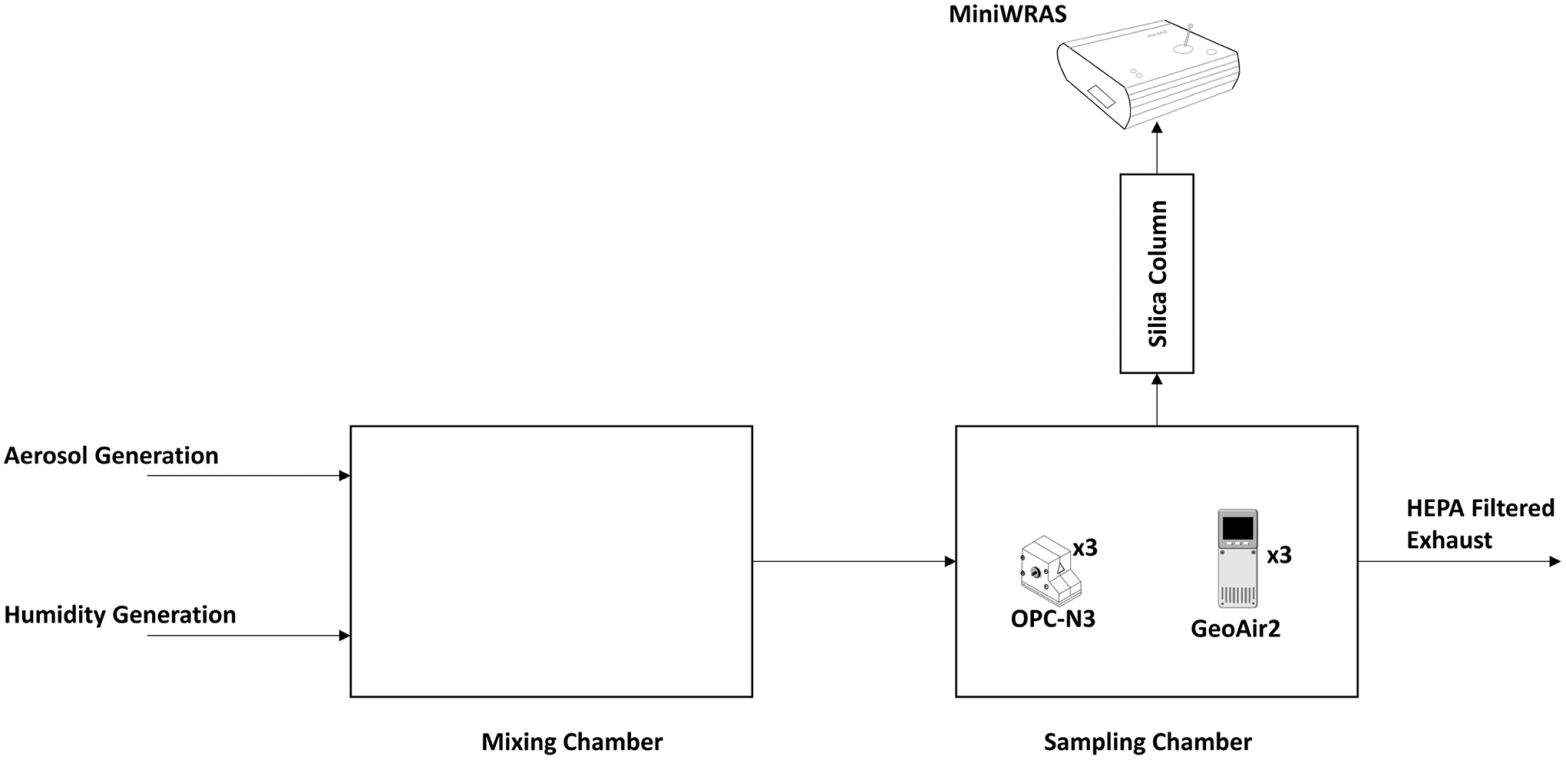
Experimental chamber designed to test the effects of different relative humidity levels on sensor reading of particulate mass.

The chamber used for the accuracy and bias assessment was too large to perform and control a humidity experiment. Therefore, smaller chambers were used for the humidity test. The relative humidity experiments were performed within two controlled, airtight Polyvinyl Chloride chambers measuring 0.36 m × 0.30 m × 0.22 m (L × W × H) and connected by a 9.5 mm hose. Positive pressure was used to move air from the mixing chamber into the sampling chamber, as shown in Fig. 2. The mixing chamber featured two inlets: a first inlet for humidified air regulated by a Miller-Nelson control system (Miller Nelson Analytical HCS-501, Brentwood, California, USA) and a second inlet connected to an aerosol generator. The sampling chamber featured a single inlet and a single outlet that led to a HEPA filter. The GeoAir2 and OPC-N3 were placed inside the sampling chamber, while the MiniWRAS sampling probe was connected to a silica drying column which was then attached to an outlet in the sampling chamber. The objective was to compare the low-cost sensor measurements with dry particle concentrations measured by the MiniWRAS since all instruments are optical sensors and are affected by humidity changes. Three pairs of GeoAir2 and OPC-N3 were used in the humidity tests considering space constraints within the chamber, exposing each to the generated aerosol.

#### 2.4.2 Aerosol generation and humidity change

Salt aerosol was generated using a Collison nebulizer (CH Technologies, Westwood, NJ, USA). The nebulizer was operated using the same mass flow controller mentioned above with a 2% (by wt.) solution of NaCl. Sensors were operated in particle-free air to achieve a 0 μg m^−3^ steady-state concentration for five minutes. Steady-state concentrations of salt aerosol were achieved at 25, 50, 75, and 100 μg m^−3^ across five relative humidity levels of 30, 50, 70, 80, and 90% with the Miller-Nelson. These steady-state concentrations were held for five minutes and monitored in real-time with the MiniWRAS.

### 2.5 Field Deployment in Indoor Residential Environments

Indoor exposure constitutes a different range of aerosol sources: cigarettes and electronic cigarettes, cooking oil, burning wood, incense, and possible outdoor sources such as dust and wood smoke (Kulkarni *et al*., 2011). Therefore, the study team recruited 3 participants to deploy air monitors in their homes in partnership with the Association of Mexicans in North Carolina (Greenville, NC). Indoor air quality was monitored for 5 days at three different homes. We recruited individuals who spend the majority of their time inside their homes because 1) daily activities they undertake at home (e.g., cooking) would allow the study team to obtain both low and high concentration data and 2) the study team had to visit their home every day during the study to change the filter in the pDR-1500 every 24 hours. A single GeoAir2 unit was placed alongside a field blank filter and a pDR-1500 equipped with a cyclone for measuring PM_2.5_ and a 37-mm fiberglass filter (Whatman, CATNon.1827–037, Maidstone, United Kingdom) for gravimetric analysis. The sampling location within the home was chosen based on the vicinity of regularly occupied living space and household activities such as cooking, where the kitchen was open to the living room. These locations were set upon tables in the living room, giving the devices a height comparable to a seated person. The living room next to an open kitchen for each house was chosen to quantify the exposure for those seated in the living area from activities in the kitchen. Photographs were not taken inside the homes to protect the privacy of the residents.

### 2.6 Data Analysis

#### 2.6.1 Accuracy, bias and precision

PM_2.5_ data for 32 GeoAir2 devices were averaged over one-minute and time-paired to create representative tables and figures for each dataset and compared directly to the MiniWRAS reference device alongside the OPC-N3 and pDR-1500. The average measurement from the 32 GeoAir2 and 6 OPC-N3’swere analyzed to determine slope, intercept, correlation coefficient (r), coefficient of determination (r^2^), Bias and coefficient of variation (CV). The average measurements were compared to the MiniWRAS reference device for both data sets, salt and ARD aerosols. The statistics were calculated for both low and high concentration data. The MiniWRAS data were filter-corrected by calculating the correction factor, the filter mass concentration divided by the average MiniWRAS real-time measurements. After this analysis, a comparison was made to EPA and National Institute for Occupational Safety and Health (NIOSH) acceptance criteria which include a slope of 1.0 ± 0.1, an intercept of 0 ± 5 μg m^−3^ (EPA), r ≥ 0.97, a bias percentage of ± 10% (NIOSH), and CV values up to 10% (EPA) (NIOSH, 2012; U.S. EPA, 2016; Sousan *et al*., 2016b, 2021). Bias and CV values were calculated using the following equations:

(1) % Sensor Bias=(Sensor−MiniWRAS)/MiniWRAS×100

(2) CV=Standard Deviation/Sensor

where Sensor is the average value reported by the low-cost sensors for the given minute of steady-state. MiniWRAS is the filter-corrected value reported by the MiniWRAS for the given minute of steady-state. Standard Deviation is the standard deviation between the individual sensors.

#### 2.6.2 Slope analysis

For each experiment, for low and high concentrations of salt and ARD aerosol, 50% of the sensors were chosen that fall within the mean slope value to identify possible sensors that can be calibrated using one correction factor (slope value) for all experiments. Finally, we identified the sensors that can be chosen for field deployment using one calibration slope factor if the slope values fall within less than a Z% of the mean slope for at least three experiments. The Z value is determined by slopes derived from the GeoAir2. Finally, a normal distribution curve was created to collectively compare the range of slope values reported from the salt and ARD experiments for low and high concentrations.

#### 2.6.3 Humidity correction

The reported concentrations of PM_2.5_ by the low-cost sensors were averaged between the achieved steady states to provide a mean value that could then be compared to the MiniWRAS reference device. SPSS was used to analyze variance and a Tukey post hoc test to derive regression equations between humidity and mass concentrations.

#### 2.6.4 Field evaluation

After deployment, pDR-1500 real-time data was filter-corrected, similar to the MiniWRAS. In addition, a time-series plot was created for the GeoAir2 data with the filter-corrected pDR-1500 data for the three-home deployments. The slope, intercept, and r^2^ values were calculated for each home.

## 3 RESULTS AND DISCUSSION

### 3.1 Accuracy, Bias and Precision

Accuracy results between the three instruments compared to the MiniWRAS reference device are shown in Table 2 for salt and ARD aerosols. During salt generation, the GeoAir2, OPC-N3, and pDR-1500 maintained a high correlation with the MiniWRAS reference device, suggesting a linear regression, although slope variation remained high between devices. Both low-cost sensors met acceptance criteria based on intercept and r value for low concentrations of salt and ARD aerosols. However, the only device that fully met EPA acceptance criteria of the slope, intercept, and r value was the GeoAir2 during low ARD aerosol concentrations, though it did not meet NIOSH bias criteria. Bias calculations suggest that the GeoAir2 consistently underestimated both aerosols and concentrations, while the OPC-N3 overestimated salt aerosol. These findings remained consistent with previous findings in Sousan *et al*. (2021), with correlation values remaining greater than 0.96. Nguyen *et al*. (2021) also produced a similar result, an r^2^ of 0.95 for the SPS30 PM_2.5_ measurements.

**Fig. 3.**
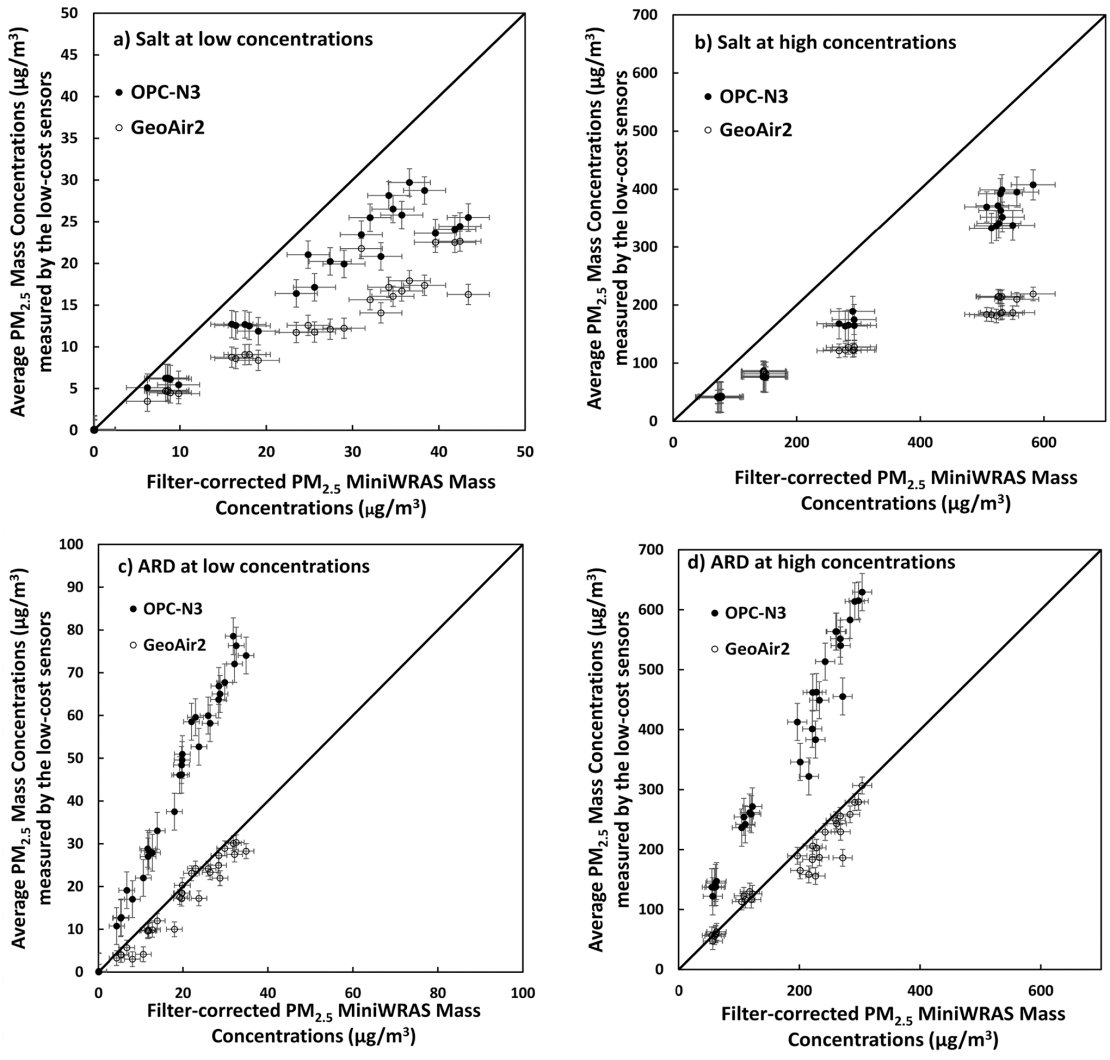
The average PM_2.5_ mass concentrations measurements by the low-cost sensors compared to the filter-corrected MiniWRAS measurements for (a) salt at low concentrations, (b) salt at high concentrations, (c) ARD at low concentrations, and (d) ARD at high concentrations. Relative humidity (35 ± 5%) and temperature (23 ± 2°C) were at normal room conditions.

The scatter plots for the average low-cost PM_2.5_ measurements compared to the filter-corrected MiniWRAS PM_2.5_ measurements for low and high salt concentrations are shown in Fig. 3. Salt aerosol exposure showed a distinct tendency to underestimate in both low-cost sensors compared to the MiniWRAS reference device. However, the OPC-N3 showed to underestimate less severely than the GeoAir2. This trend was visible in low and high concentrations of salt aerosol, with sensors tending to underestimate. These results were consistent with previous findings in Sousan *et al*. (2021), where readings from the OPC-N3 during PM_2.5_ salt aerosol experiments were underestimated compared to the MiniWRAS reference device but less so than for the GeoAir2. Sousan *et al*. (2021) observed that the SPS30 slightly overestimated PM_2.5_ concentrations compared to non-filtered MiniWRAS concentrations, and underestimated PM_2.5_ concentrations compared to filtered MiniWRAS concentrations. Therefore, this indicates that the raw GeoAir2 data are comparable to the raw MiniWRAS data, and these results are affected by the salt filter correction factor. The OPC-N3 has been known to underestimate during experiments with other aerosols, including welding fumes which are fine particles (Sousan *et al*., 2016a).

The scatter plots for low and high ARD concentrations showed a tendency of overestimation by the OPC-N3 compared to the MiniWRAS reference device (Fig. 3), which is consistent with previous findings in Sousan *et al*. (2021). The GeoAir2 monitor was significantly closer to the MiniWRAS reference device during ARD generation than the salt generation, though it was slightly underestimated. These results yielded a linear relationship between the GeoAir2 and the MiniWRAS. This result is slightly different from the one in Sousan *et al*. (2021), which concluded that SPS30 significantly underestimated concentrations of ARD compared to the MiniWRAS. However, the results of the current study are more reliable because this study tested 32 units of SPS30 sensors, while 3 units were tested in the previous study. The findings of this study suggest that the GeoAir2 is a more suitable platform for measuring indoor dust aerosol than the OPC-N3.

### 3.2 Slope Analysis

Comparing the reported slope values of each GeoAir2 device to the mean slope of each respective experiment showed that only 19 devices met the criteria three or more times. Therefore, an interval of Z = 20% was chosen to allow at least half of the devices to report at least two mean slopes within the acceptable threshold. The mean slopes for low and high concentrations of salt and ARD were 0.53, 0.33, 0.98, and 0.8, respectively. Similar results have been produced in a larger-scale calibration using Sharp low-cost sensors (GP2Y1010AU0F, Sharp Electronics, Osaka, Japan) (Sousan *et al*., 2018).

**Fig. 4.**
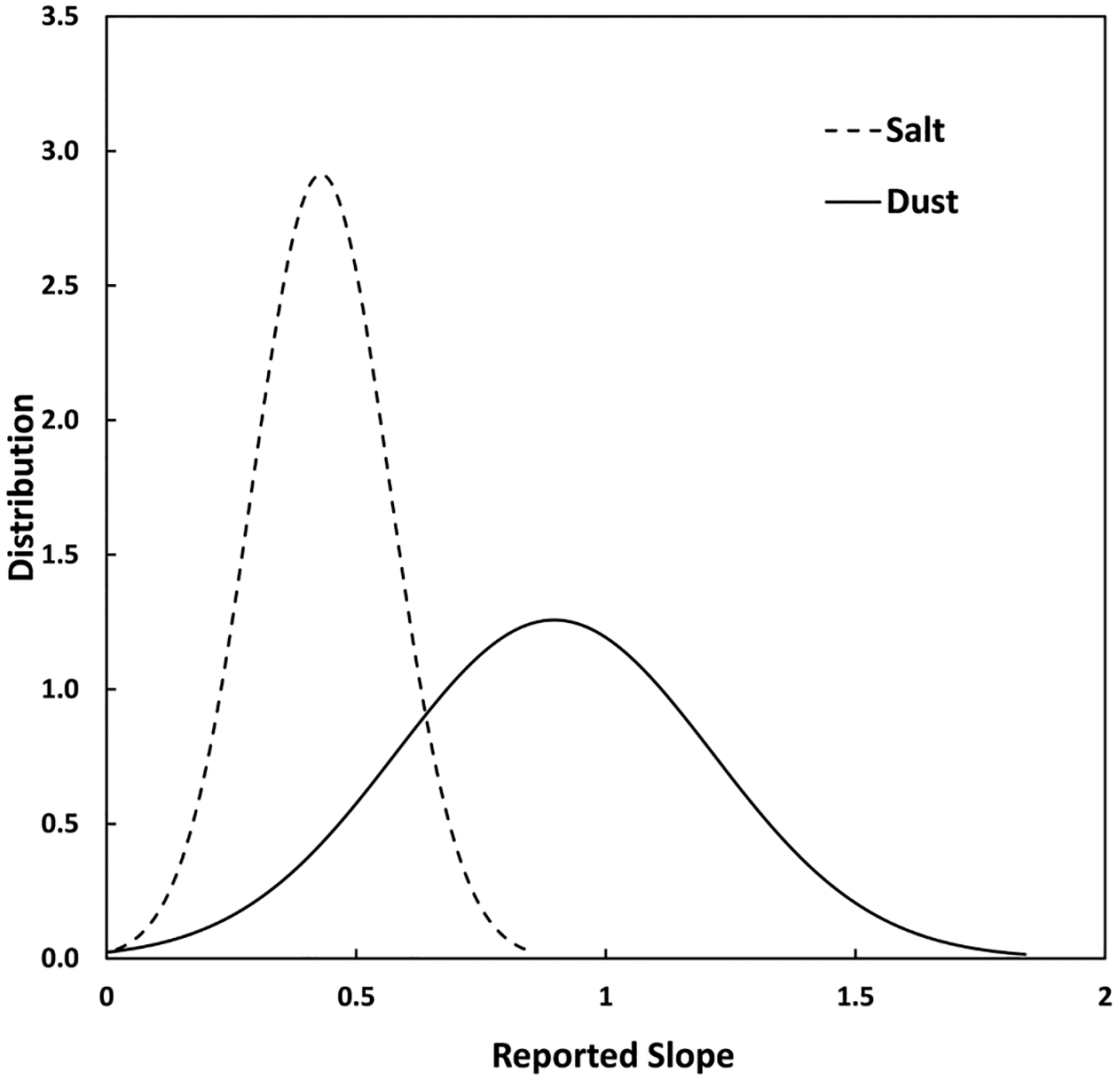
The normal distribution of slopes was recorded by the GeoAir2 units across all salt aerosol testing.

The salt and ARD normal distributions for all the slopes, low and high concentrations, of the GeoAir2 are displayed in Fig. 4. These results show that when the GeoAir2 was calibrated with salt, all the sensors underestimated the true concentrations for low and high concentrations. In contrast, when the GeoAir2 was calibrated with ARD, 53% of the sensors underestimated the low concentrations and 75% underestimated the high concentrations. Therefore, the range of slopes reported for salt aerosol was narrower than for ARD aerosol. It may be because the manufacturer calibrates the SPS30 with salt aerosol (Sousan *et al*., 2021). In addition, the results show that calibration by one aerosol cannot be considered universal for other aerosols. These results are similar to other studies that have shown that calibration equations are dependent on aerosol type (Wang *et al*., 2015; Sousan *et al*., 2016a, 2016b). Therefore, calibration would be best performed on-site with the aerosols expected. This unexpected variation in slope may affect the deployment of sensors based on calibration by non-target aerosol for indoor use.

### 3.3 Humidity Correction

**Fig. 5.**
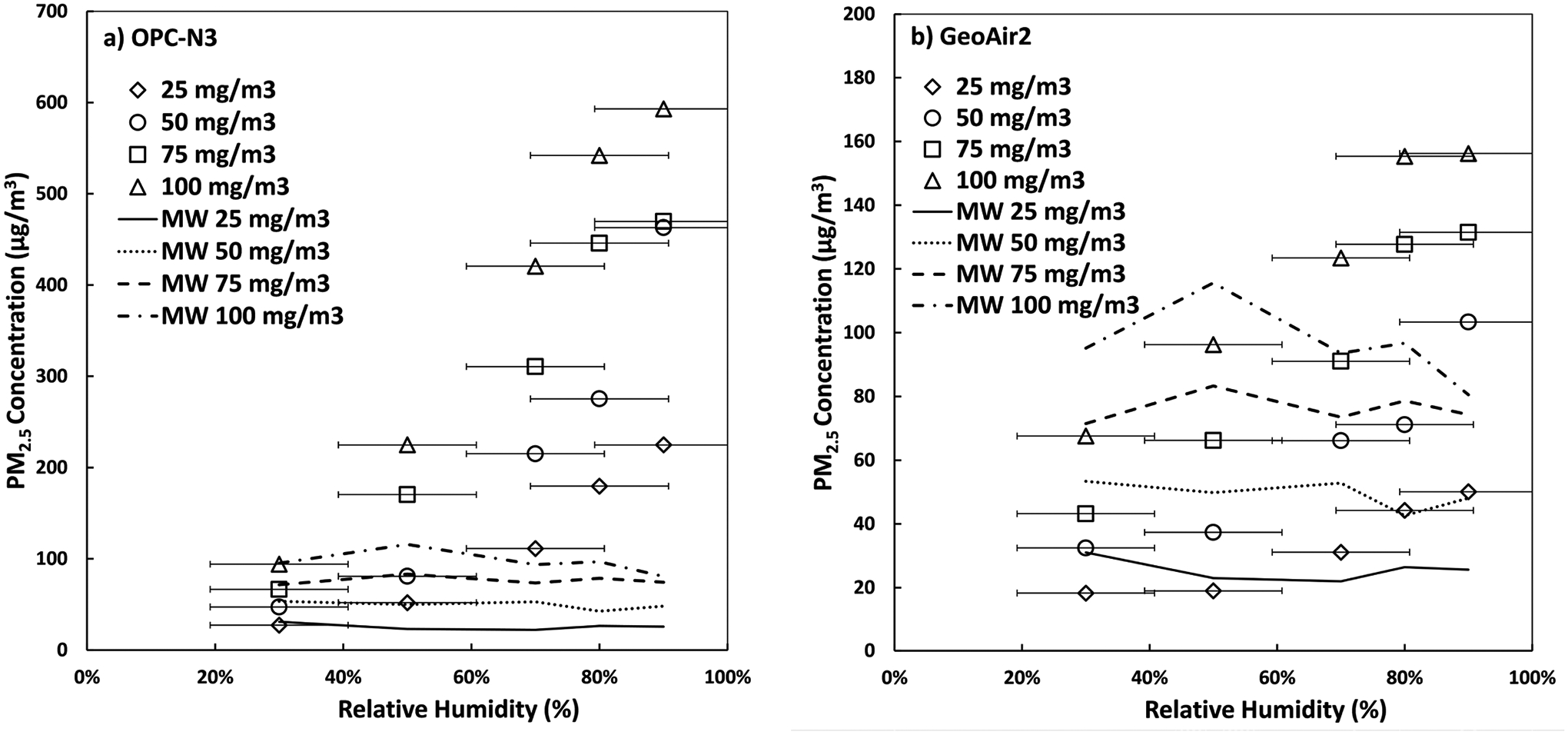
Relative humidity (%) effects on PM_2.5_ concentrations (μg m^−3^) detected by the (a) OPC-N3 and (b) GeoAir2 compared to the MiniWRAS (MW) PM_2.5_ concentration at different steady states of salt aerosol. The MW was not affected by the humidity because the particles were dried before measurements were performed.

PM_2.5_ mass concentrations of the OPC-N3 and GeoAir2 compared to the MiniWRAS at different humidity levels are shown in Fig. 5. For the OPC-N3, humidity effects were large, where PM_2.5_ mass concentrations increased 425% for low concentrations (25–50 μg m^−3^) and 181% for high concentrations (75–100 μg m^−3^) when the humidity changed from 50% to 90%, respectively. For the GeoAir2, the humidity effects were much lower, where PM_2.5_ mass concentrations increased 100% for low concentrations and 137% for high concentrations when the humidity changed from 50% to 90%, respectively. The change in relative humidity increased the overestimation for both low-cost sensors due to hygroscopic growth in both low and high concentrations. The increase in magnitude was also found in another study (Zou *et al*., 2021). This level of hygroscopic growth was also observed for the SPS30 and OPC-N3 in Wang *et al*. (2021).

Humidity correction equations were developed for the OPC-N3 and GeoAir2:

(3) $$\text{OPC-N}3{\text{ Measured PM}}_{2.5}\left(\mu {\text{g m}}^{-3}\right)=-371.13+3.38\text{Concentration}+6.42\text{Humidity }\left({\text{R}}^{2}=0.91\right)$$

(4) $$\text{GeoAir}2{\text{ Measured PM}}_{2.5}\left(\mu {\text{g m}}^{-3}\right)=-73.26+1.17\text{Concentration}+1.20\text{Humidity }\left({\text{R}}^{2}=0.93\right)$$

where the Concentration is a steady-state concentration achieved (range 25–100 μg m^−3^) and Humidity is relative humidity achieved (range 30 to 90%). These results show that the GeoAir2 device is less affected by the change in relative humidity than the OPC-N3. The data suggest that compared to the OPC-N3, the GeoAir2 is not only a better option for indoor settings, but also a better option for use in environmental conditions where the relative humidity is expected to be high or variable such as outdoor settings.

### 3.4 Field Evaluation

**Fig. 6.**
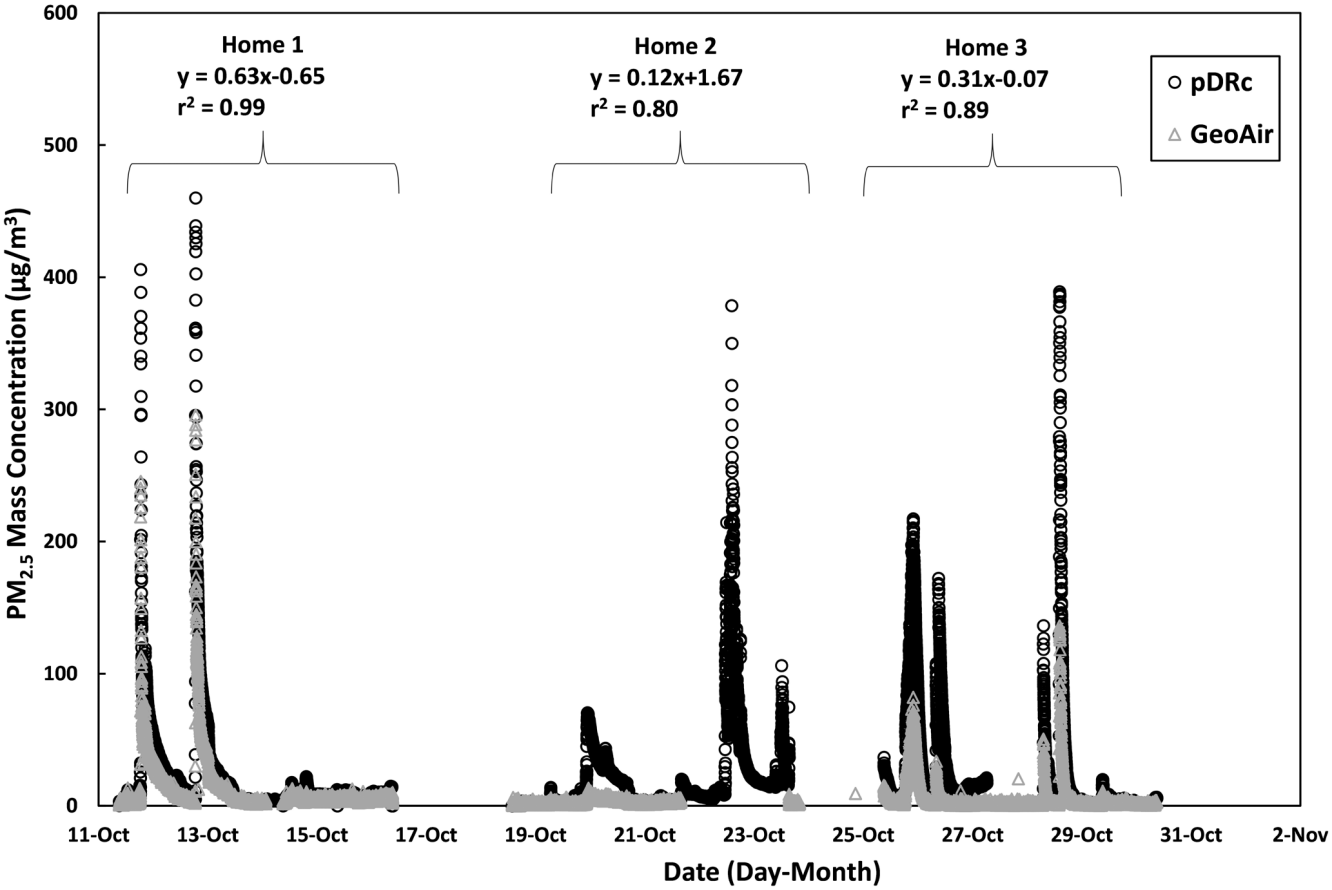
Time series plot of the filter-corrected pDR-1500 measurements and GeoAir2 measurements in three different indoor residential environments.

The time series plot of the pDR-1500 and GeoAir2 measurements in indoor settings is shown in Fig. 6. The Home 2 plot represents 4-day data because the GeoAir2 failed to log for one day. There was also a loss of data for Home 3 because the pDR-1500 lost power for one day. The pDR-1500 data were corrected with the filter measurements when the filters were above the limit of detection of 0.50 mg. The r^2^ values were lower in Homes 2 and 3, which may be related to the loss of data and the smaller sample size. Therefore, it is expected that the presence of this data would have increased the r^2^ values to levels comparable to the r^2^ value of 0.99 for Home 1. The increase of side-by-side sampling time has been shown to increase correlation, as suggested by Sousan *et al*. (2018). These results provide a higher correlation than those found by Demanega *et al*. (2021), with slopes of indoor PM_2.5_ concentrations compared to the estimated true concentration ranging from 0.22 to 0.82. The full set of data collected at Home 1 indicates that the high correlation between readings from the GeoAir2 and the filter-corrected pDR-1500 suggests that the GeoAir2 is a promising low-cost sensor for residential use. Future work will test GeoAir 2 in non-residential, indoor settings.

Optical sensors are affected by aerosol type where the sensor performance differs based on aerosol refractive index, particle size and shape. Previous studies have discussed the difference between aerosol types and optical sensor performance (Sousan *et al*., 2016a, 2016b). These studies emphasized that the performance of optical sensors is considerably superior for non-light-absorbing particles such as salt and dust when compared to light-absorbing particles such as diesel and welding fumes. Therefore, the GeoAir2 sensor would be suitable for measuring aerosols produced from cigarettes, cooking oil, incense, and dust, compared to soot particles from burning wood.

## 4 CONCLUSIONS

This study found that the GeoAir2 performed better than the higher-cost OPC-N3 for indoor environments. In laboratory settings, the correlation with the MiniWRAS remained high across both salt and ARD measurements. In addition, the GeoAir2 was less influenced by changes in humidity when compared to the OPC-N3 during salt aerosol experiments. In indoor residential environments, the GeoAir2 was highly correlated with the filter-corrected pDR-1500. These findings suggest that the GeoAir2 is more suitable for indoor environments than the OPC-N3 due to the more accurate results and lessened effect of changing environmental conditions (humidity). Therefore, the GeoAir2 shows great potential for indoor air quality monitoring and exposure assessments when calibrated on-site. Future work will focus on the outdoor environmental evaluation of the GeoAir2.

## Figures and Tables

**Table 1.** Specifications of the low-cost and reference monitors.

Technical Data	GeoAir2	OPC-N3	pDR-1500	MiniWRAS
Cost ($)	300	500	7,000	30,000
Size range (μm)	0.30–10.00	0.30–40.00	One size based on cyclone	0.01–35.00
Type (active or passive flow)	Active	Active	Active	Active
Bin size (software bins, dimensionless)	5	24	-	41
Concentration range (μg m^−3^)	0–1,000	0–2,000	0.001–400,000	0–100,000
Mass concentration measurement	PM_1_, PM_2.5_, PM_10_	PM_1_, PM_2.5_, PM_10_	PM_1_, PM_2.5_, PM_10_	PM_1_, PM_2.5_, PM_10_
Sampling flow rate (L min^−1^)	-	-	1.52	1.20
Number concentration	Yes	Yes	No	Yes
Sampling frequency	1 s	1 s	1 s	1 min
Internal rechargeable battery	Yes	No	Yes	Yes
Dimensions: L × W × H (m)	0.055 × 0.140 × 0.0375	0.075 × 0.006 × 0.060	0.181 × 0.143 × 0.0484	0.34 × 0.31 × 0.12

**Table 2.** Evaluation of average correlation factors across measuring devices compared to the MiniWRAS reference device during tests involving salt and ARD aerosols at low and high concentrations^a^. Relative humidity (35 ± 5%) and temperature (23 ± 2°C) were at normal room conditions.

Concentration	Instrument	No. of Samples	Slope	Intercept	r	r^2^	%Bias	%CV
Salt Low Concentration	pDR	36	1.12	0.42	0.99	0.98	14.47	
	OPC-N3	36	0.67	0.82	0.98	0.86	−28.10	39.82
	GeoAir2	36	0.53	−0.46	0.96	0.93	−49.34	20.58
Salt High Concentration	pDR	36	0.66	11.10	0.97	0.94	−25.17	
	OPC-N3	36	0.72	−24.33	0.99	0.99	−37.78	49.50
	GeoAir2	36	0.34	20.23	0.98	0.97	−51.52	20.16
ARD Low Concentration	pDR	36	1.67	−2.58	0.99	0.99	51.53	
	OPC-N3	36	2.33	0.13	0.99	0.99	134.83	41.47
	GeoAir2	36	0.99	−2.86	0.99	0.99	−18.67	37.63
ARD High Concentration	pDR	36	1.48	18.17	0.98	0.97	64.11	
	OPC-N3	36	1.91	27.93	0.99	0.98	112.40	41.00
	GeoAir2	36	0.85	6.71	0.99	0.98	−9.24	38.74

a The low concentrations are considered to be 0 to 50 μg m^−3^ based on EPAs PM_2.5_ standard for 35 μg m^–3^, while high concentrations are considered to be 50 to 500 μg m^−3^ in this experiment.
